# Social feedback interferes with implicit rule learning: Evidence from event-related brain potentials

**DOI:** 10.3758/s13415-018-0635-z

**Published:** 2018-09-06

**Authors:** Philippa J. Beston, Cécile Barbet, Erin A. Heerey, Guillaume Thierry

**Affiliations:** 1 School of Psychology, Bangor University, Brigantia Building, Penrallt Road, Bangor, Wales LL57 2AS UK; 2 Faculty of Psychology and Educational Sciences & Department of Linguistics, University of Geneva, Geneva, Switzerland; 3 Psychology Department, Western University, London, Ontario Canada

**Keywords:** Implicit learning, Social feedback, Event-related potentials, P3b, fERN

## Abstract

The human brain can learn contingencies built into stimulus sequences unconsciously. The quality of such implicit learning has been connected to stimulus social relevance, but results so far are inconsistent. We engaged participants in an implicit-intentional learning task in which they learned to discriminate between legal and illegal card triads on the sole basis of feedback provided within a staircase procedure. Half of the participants received feedback from pictures of faces with a happy or sad expression (social group) and the other half based on traffic light icons (symbolic group). We hypothesised that feedback from faces would have a greater impact on learning than that from traffic lights. Although performance during learning did not differ between groups, the feedback error-related negativity (fERN) was delayed by ~20 ms for social relative to symbolic feedback, and the P3b modulation elicited by infrequent legal card triads within a stream of illegal ones during the test phase was significantly larger in the symbolic than the social feedback group. Furthermore, the P3b mean amplitude recorded at test negatively correlated with the latency of the fERN recorded during learning. These results counterintuitively suggest that, relative to symbolic feedback, socially salient feedback interferes with implicit learning.

Humans can learn contingencies about their environment without conscious awareness. Such phenomenon is classically observed in the case of statistical learning, when dependencies between linguistic stimuli, for instance, are extracted by the brain without the participants’ intention to acquire them (Saffran, Aslin, & Newport, 1996). Such form of spontaneous and unconscious learning is observed across a variety of perceptual domains. In the auditory domain, for example, studies have shown that infants implicitly use language patterns to rapidly segment words from speech streams (Aslin, Saffran, & Newport, 1998; Saffran, 2003), and this phenomenon extends even beyond linguistic stimuli (Saffran, Johnson, Aslin, & Newport, 1999). Statistical learning occurs spontaneously (Fiser & Aslin, 2001), rapidly (Turk-Browne, Scholl, Chun, & Johnson, 2009), and without the need for explicit instruction (Fiser & Aslin, 2002). This has led to the proposal that statistical learning results in the formation of implicit knowledge (Fiser & Aslin, 2002; Turk-Browne, Jungé, & Scholl, 2005; Perruchet & Pacton, 2006; Reber, 1967).

Implicit learning refers to the process of learning the underlying rule of a system (e.g., an artificial grammar) solely based on exposure to stimulus contingencies and probabilities (Reber, 1967). Just like statistical learning, implicit learning is thought to be unconscious, meaning that participants are unable to verbalise a rule that they have acquired (Reber, 1989) and are not aware that they have learnt something (Cleeremans, Destrebecqz, & Boyer, 1998; Dienes, Altmann, Kwan, & Goode, 1995; Seger, 1994). For example, evidence from the serial reaction time task suggests that people identify previously viewed light sequences more quickly than novel sequences (Willingham, Nissen, & Bullemer, 1989; Chun & Jiang, 1998).

One limitation of much work within this literature is that the nature and quality of learning is measured by participant performance or metacognitive evaluations after learning. In the problem-solving domain, for example, implicit memory of a puzzle improves problem solving on a subsequent task, even when participants are given a concurrent task to exert strain onto the working memory system (Reber, 1989; Reber & Kotovsky, 1997). The results of such studies appear to be contingent upon the type of task used to determine whether learning was implicit (Shanks & Channon, 2002; Wilkinson & Shanks, 2004). Thus, the extent to which the process is truly unconscious remains debatable. Still, when participants perform above chance after training, although they believe that they are merely guessing the answers, one may presume that the learning was mostly unconscious and that their knowledge is implicit (Dienes et al., 1995). Shanks and St. John (1994) have questioned how much post-learning tests (e.g., asking participants to verbalise a rule that they have acquired) tell us about the nature of the learning process. More specifically, they enquired whether tests of performance and awareness are sensitive enough to measure the acquired knowledge that has become conscious and whether knowledge awareness can really be assessed before the nature of the knowledge itself has been determined. It seems that classic implicit learning tasks lack precision regarding the nature of what participants learn when the conclusions are solely drawn from performance indices, e.g., reaction time (Eimer, Goschke, Schlaghecken, & Stürmer, 1996) or post-learning verbalisations (Shanks & St John, 1994).

One way to obtain unbiased evidence of implicit learning is to measure spontaneous brain activity modulations elicited by learned contingencies. Event-related potentials (ERPs), a method derived from electroencephalography, are averaged recordings of brain activity measured at the surface of the scalp elicited by series of repeated stimuli. ERPs can be recorded independently of performance indices and index unconscious information processing in the absence of any behaviourally measurable effect (Thierry & Wu, 2007; Wu & Thierry, 2010). Baldwin and Kutas (1997) showed that participants engaged in an artificial grammar learning task (without any explicit instruction regarding underlying rules) produced P300 ERP responses of larger amplitude for correct grammatical forms than incorrect ones. This result shows that participants developed expectancies about the sequences they viewed and were able to detect rule violations, even though they seemed unable to consciously access this information at debriefing (Van Zuijen, Simoens, Paavilainen, Näätänen, & Tervaniemi, 2006). Similar effects have even been shown in cases where rule learning was not embedded within the experiment but rather occurred from natural exposure to language. For example, Vaughan-Evans et al. (2016) recently showed that the brains of individuals with no recorded or overt knowledge of an ancient form of Welsh poetry (Cynghanedd) successfully identified correct forms from sentences violating composition rules, despite being unable to detect the correct forms in overt judgement tasks. Presumably, these participants learned the rules of Cynghanedd implicitly, through natural language exposure and required no conscious knowledge.

One important dimension of the human learning environment that seems to have been neglected in the implicit learning literature is the social quality of the information people learn, even though it is reasonable to assume that feedback during learning would vary in efficiency depending on its social significance. Information from and about other humans is abundant in the environment, and even the mere presence of others has long been suggested to facilitate performance on simple tasks (Bond & Titus, 1983; Zajonc, 1965; Zajonc, Heingartner, & Herman, 1969). More recent research has suggested that reliable social cues allow others to implicitly predict their behaviour, e.g., in a game of rock-paper-scissors (Heerey & Velani, 2010). Social cues, such as smiles and frowns, can aid performance during associative learning compared with nonsocial “traffic light” feedback (i.e., “symbolic” feedback; Hurlemann et al., 2010). These findings suggest that socially relevant information is processed by the same associative system that underlies other types of reward-based learning (Behrens, Hunt, Woolrich, & Rushworth, 2008). However, during cognitively demanding tasks, participants avert gazing at faces, and the frequency of this avoidance relates to task difficulty (Doherty-Sneddon, Bruce, Bonner, Longbotham, & Doyle, 2002; Glenberg, Schroeder, & Robertson, 1998). Thus, social information appears to add a cognitive load during difficult tasks and participants spontaneously resort to gaze averting in order to reduce this load (Doherty-Sneddon & Phelps, 2005).

Nevertheless, there is a distinction between learning that occurs within a social context (Glenberg et al., 1998; Heerey & Velani, 2010) and learning that results in a socially charged outcome, e.g., when socially relevant information conveys feedback about performance (Turnbull, Bowman, Shanker, & Davies, 2014). In the latter case, there is some indication of a facilitatory effect on performance in associative tasks (Hurlemann et al., 2010); however, it is unknown how performance is affected in tasks that require implicit contingency learning. We presented participants with triads of cards featuring coloured shapes, varying in four possible ways (shape type, colour, number of shapes, and filling) and asked them to indicate which triads were “legal” combinations and which were “illegal,” according to a rule that was never described. Participants were thus engaged in an implicit-intentional learning task, in which they were instructed to proceed on a trial-and-error basis. We labeled this context as intentional, because participants were aware of the need to extract some kind of rule, even though they did not know this rule. This task context notably differs from the incidental context that usually applies in classical implicit learning. They received feedback on every trial, completing the learning phase only when they had met a predetermined learning criterion applied via a staircase procedure (described in Procedures section). Participants received feedback with either faces or a traffic light display. Given their high social-relevance and the fact they have been shown to increase performance in associative tasks (Hurlemann et al., 2010; Mihov et al., 2010), we hypothesised that faces as feedback would boost performance during learning and result in higher accuracy in a subsequent test phase. Crucially, to collect an objective and spontaneous marker of learning, we recorded ERPs throughout the two phases of the experiment and monitored: (a) the participants’ physiological reaction to feedback (indexed by the feedback error-related negativity, fERN) during the learning phase, and (b) their spontaneous response to infrequent legal card combinations, presented amongst frequent illegal ones (as indexed by the P3b modulation elicited by infrequent stimuli in an oddball paradigm) during the test phase. Consistent with our hypothesis, we expected that face stimuli would elicit greater fERN amplitudes during learning and thus lead to greater mean P3b amplitudes at test.

## Methods

### Participants

Fifty-five Bangor University students (27 females; *M*_age_ = 21.6, *SD* = 3.7) were recruited to participate via the university’s participant panel and received course credit as compensation. Of these participants, 27 (15 females; *M*_age_ = 22.4, *SD* = 4.8) received social feedback, and 28 (12 females; *M*_age_ = 20.9, *SD* = 1.9) received symbolic feedback during the learning phase of the experiment. Experimenters were blind to feedback condition when instructing participants in the learning phase. All participants provided written, informed consent to take part in the study, which was approved by the Ethics Committee of the School of Psychology at Bangor University. We excluded four individual datasets from all analyses because of excessive time spent on, or failure to complete, the learning phase (see criterion in *Procedure* below). We further excluded 4 datasets of the remaining 51 from analysis for the learning phase and a different 4 of 51 for the test phase; datasets were included on the basis of a sufficient number of trials to analyse being present. Thus, the final samples for statistical analysis in the learning phase was 47 (24 social, 13 females; 23 symbolic, 11 females) and 47 (23 social, 14 females; 24 symbolic, 11 females) for the test phase.

### Stimuli

**Fig. 1:**
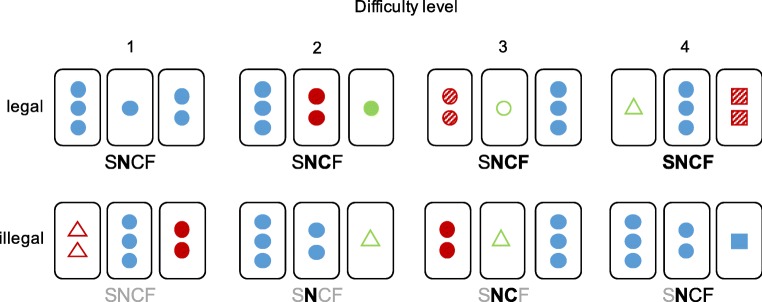
Examples of legal and illegal card triads split by levels of difficulty. Note that full repetition triads (same shape, S, number, N, colour, C, and filling, F) were not used in the experiment, because they were too simple to identify. The code under each triad indicates the particular properties that comply with the rule: black slim letters code for a dimension repeated across all three cards, black bold letters code for dimensions different across all three cards, and grey letters indicate dimensions for which the all same/all different rule is violated

Eighty-one cards each featuring a unique combination of one to three shape(s) (circle, square, triangle), in one of three colours (red, green, blue), with one of three fillings (empty, hashed, full) were used to create card triads that either complied or not with the following rule: A legal combination is a triad of cards in which all cards are the same or different, considering each of the stimulus dimensions separately (shape, number, colour, filling). Any combination of cards featuring a partial repetition of any stimulus dimension was thus illegal (Fig. 1). Card triads were further split into four difficulty levels based on the perceived difficulty in assessing legality, e.g., a combination of cards failing the all same/all different criterion for all four dimensions was considered relatively easy to spot as illegal (cf. illegal difficulty level 1 in Fig. 1).

The number of possible card combinations differed between difficulty levels (e.g., illegal level 1:1 combination, illegal level 4: 32 possible combinations). In the learning phase, the weighting of each combination was adjusted to ensure that each level had equal probability of being presented throughout the staircase procedure to allow participants to learn about combinations from all of the levels. During the test phase, however, we elected to present difficulty levels at their “natural” frequency, and legal and illegal conditions were presented with a ratio of 1:3 to comply with the oddball design.

Card triads were presented under 4 degrees of visual angle in the learning phase and under less than 2 degrees of visual angle in the test phase, that is, in participants’ foveal visual field to avoid eye saccades and consequent artefacts. Note that no ERPs were analysed in response to card triads in the learning phase.

Feedback: Twelve pictures of faces (6 females, 6 males) each presented with a happy, neutral, or sad expression were collated from The Karolinska Directed Emotional Faces database (KDEF: Lundqvist, Flykt, & Öhman, 1998; Goeleven, De Raedt, Leyman, & Verschuere, 2008) and edited to fit within 2 degrees of visual angle. Six simple shapes (circle, square, triangle, hexagon, diamond, trapezoid) were drawn to fit the same surface as that covered by faces and coloured in green, orange, or red in two different levels of luminance as a counterpart to the two genders for faces (6 dark, 6 light).

### Procedure

#### Learning phase

Participants first completed an implicit-intentional learning task. On each trial, a card triad randomly selected from a database of card combinations was presented in the middle of a 19” CRT monitor with a refresh rate of 74 Hz. Legal and illegal combinations had equal probability of presentation as did levels of difficulty. Participants had to indicate whether the current combination was “legal” or “illegal” by pressing designated buttons on an SR response box (E-Prime 2.0 software; Psychology Software Tools, Pittsburgh, PA). Participants started on a random response basis. In the symbolic group, feedback was provided by means of shapes filled in one of two colours (green, correct; red, incorrect). Thus, the symbolic feedback stimuli shared some perceptual similarity with the card stimuli (i.e., some shapes and some colours), but the colour scheme of the symbolic feedback was semantically transparent (green for correct and red for incorrect) and binary, thus entirely unambiguous whereas the shapes and colours presented on cards had no intrinsic meaning and were completely arbitrary. In the social group, participants received feedback from pictures of faces with one of two different emotional expressions (smile, correct; sad, incorrect).

Before the feedback stimulus, a neutral stimulus (orange shape in the symbolic group and neutral face in the social group) was displayed with a pseudorandom variable duration (750-950 ms in steps of 40 ms). The neutral stimulus served to focus the participant’s attention in the centre of the screen (thus avoiding eye movements) and desynchronised the fERN response elicited by the subsequent valenced feedback stimulus from the ERP elicited by the card triad.

**Fig. 2:**
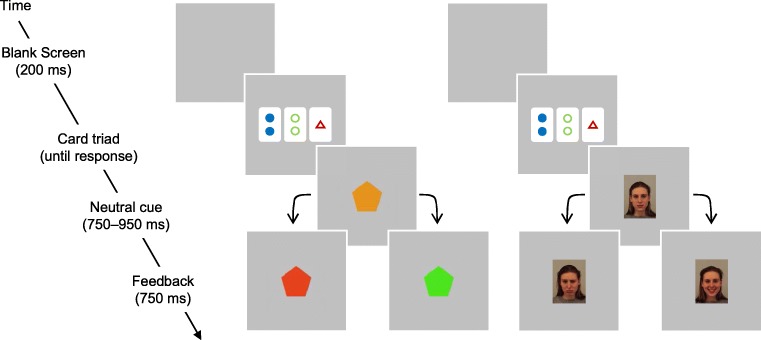
Structure of a trial on the staircase learning procedure. After presentation of the card triad, participants received one of two types of feedback: symbolic (left) or social (right), preceded by a neutral stimulus in all cases

Participants progressed through a staircase procedure such that they had to make five correct cumulative judgements for each level of difficulty in each legal and illegal condition before triads from that level and condition were dismissed from training (Fig. 2). Any error reset the count of correct trials to zero for the current level of difficulty and condition. Response side was counterbalanced across participants.

#### Testing phase

**Fig. 3:**
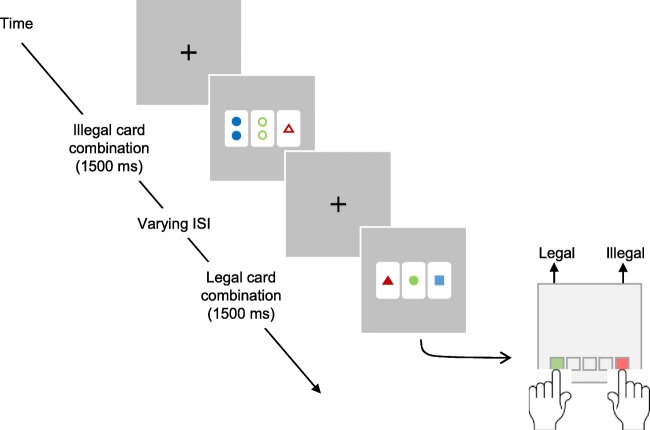
Trial structure of the test phase. Participants were required to respond on every trial using the SR box provided. Response side counterbalanced across participants

After completing the learning phase, participants were asked in a second phase to indicate whether each card triad presented was legal or illegal without feedback. Each triad was presented for a maximum duration of 1,500 ms and response initiated the next triad presentation after an inter-stimulus interval of 450-550 ms (in steps of 25 ms) during which a fixation cross then appeared in the centre of the screen. The random interstimulus interval was introduced to reduce cross-trial ERP contamination (Fig. 3). There were three blocks of 200 trials and participants were given the chance to rest between each block. Legal trials were presented with an average frequency of 25% (range 23–26%) and were expected to act as deviants amongst frequent illegal triads, thus conforming to the structure of a classic oddball paradigm prone to eliciting P3b ERP effects.

### EEG recording and analysis

EEG data were recorded continuously at a sampling rate of 1 kHz in reference to Cz using 64 Ag/AgCl electrodes attached to an elastic cap (Easycap™, Herrsching, Germany) and arranged according to the extended 10-20 convention. EEG signals were amplified using SynAmps2™ (Neuroscant™ Inc., El Paso, TX). The ground electrode was placed at FPz. Four additional electrodes were placed to the right of the right eye and to the left of the left eye (HEOG) and above and below the right eye (VEOG) to monitor horizontal and vertical eye movements. Impedances were kept below 5 kΩ. Recordings were filtered online between 0.01 and 200 Hz (slope 24 db/Oct.).

The EEG data were filtered offline using a zero-phase shift bandpass digital filter between 0.1 Hz [24 db/Oct]–25 Hz [48 db/Oct] using Scan 4.5 (Neuroscan™ Inc.). Major artefacts were manually rejected, and eye blinks were mathematically corrected according to the procedure described in Gratton, Coles, and Donchin (1983). Continuous EEG activity was then segmented into epochs ranging from 100 to 1000 ms after stimulus onset for the learning phase and −200 to 1,000 ms for the testing phase. A shorter baseline window was selected in the learning phase to minimise baseline contamination by the preceding neutral stimulus cue. Baseline correction was performed in reference to prestimulus activity, and individual averages were digitally re-referenced to the global average reference.

In the learning phase, the average number of feedback trials included in the symbolic condition was 43.48 (SEM = 4.64) and 45 (SEM = 8.20) in the social condition. As for the test phase, only accurate trials were kept for the analysis, leading to an average of 381.54 (SEM = 12.97) trials in the symbolic condition and 346.48 (SEM = 14.89) trials in the social condition.

The ERP modulation of interest in the learning phase was the feedback error-related negativity (fERN), which is typically maximal over frontocentral electrodes and typically peaks between 200-320 ms (Ma, Meng, & Shen, 2015; Miltner, Braun, & Coles, 1997; Scheffers & Coles, 2000). We thus analysed fERN mean amplitude at FC1, FCz, FC2, C1, Cz, and C2 between 200–320 ms—the predicted time-windows based on previous studies. fERN peak latency was fixed in each condition and each participant and measured at the electrode of minimum amplitude FCz where the fERN was most negative (Picton et al., 2000). As for the test phase, rare legal stimuli were expected to elicit larger P3b amplitudes than frequent illegal stimuli. P3b mean amplitudes were analysed over the predicted centroparietal region (C1, Cz, C2, CP1, CPz, CP2, P1, Pz, P2) between 480-580 ms where it is classically analysed in tasks requiring elaborate cognitive processing (Kok, 2001; Polich, 2007).

### Statistical analyses

Behavioural and ERP results were analysed using mixed design ANOVAs with legality (illegal, legal) as repeated-measures factors and feedback condition (social, symbolic) as a between-groups factor. Greenhouse-Geisser corrections were applied when necessary, and *df* and *p* values reported are adjusted.

## Results

### Learning phase

#### Performance

Because speedy responses were not encouraged, we did not analyse reaction times in the learning phase. Analysis of accuracy showed a main effect of legality, F(1,49) = 15.93, *p* < 0.001, ${\upeta}_{\mathrm{p}}^2$ = 0.245, but no main effect of feedback condition or interaction (*p* ≥ 0.819). Participants responded to legal combinations significantly more accurately than illegal ones (*M*_Legal_ = 0.73, *SD* = 0.12; *M*_Illegal_ = 0.66, *SD* = 0.10).

#### fERN

In order to analyse the fERN, a difference waveform was computed by subtracting the grand average positive feedback waveform from the negative feedback waveform (Miltner et al., 1997; Scheffers & Coles, 2000). There was no main effect of legality or feedback condition on mean fERN amplitude, nor significant interaction between the two (*p* ≥ 0.228).

**Fig. 4:**
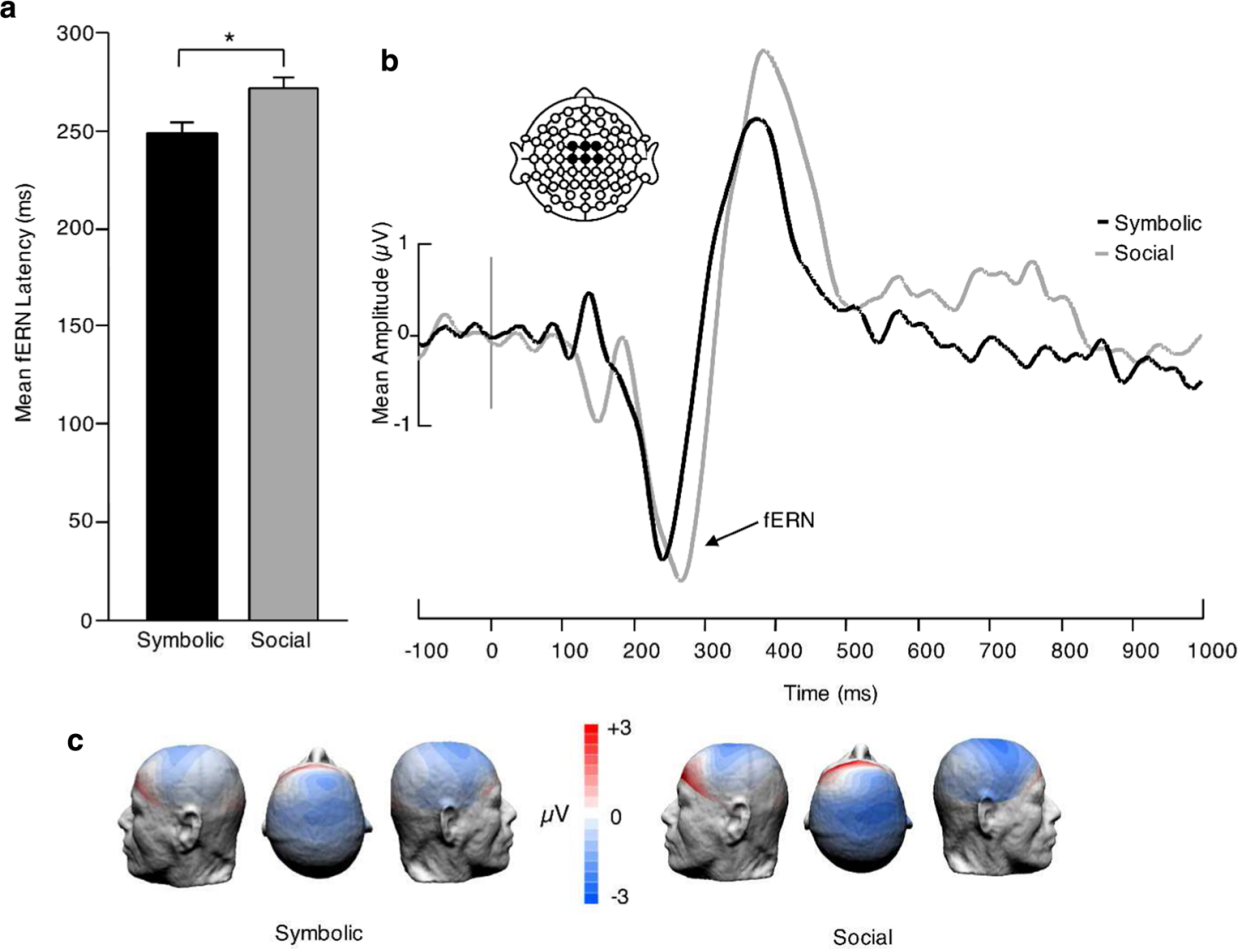
Effect of feedback condition on the fERN (negative minus positive feedback). (**a**) fERN mean latencies by feedback condition. (**b**) Grand-average ERP difference waveforms elicited over the frontocentral region (linear derivation of FC1, FCz, FC2, C1, Cz, and C2) in the symbolic (black line) and social (grey line) conditions. (**c**) fERN topographies (200-320 ms) by feedback condition. *p < 0.05

However, there was a significant main effect of feedback condition on fERN peak latency, F(1,45) = 5.879, *p* = 0.019, ${\upeta}_{\mathrm{p}}^2$ = 0.116. There was no main effect of legality or interaction between legality and feedback condition (*p* ≥ 0.560). Therefore, we collapsed mean latencies across legality and tested feedback condition using an independent samples *t* test and found a significant difference, *t*(45) = −3.08, *p* = 0.004, *d* = 0.90, confirming the previous result (Fig. 4).

### Test phase

#### Performance

**Fig. 5:**
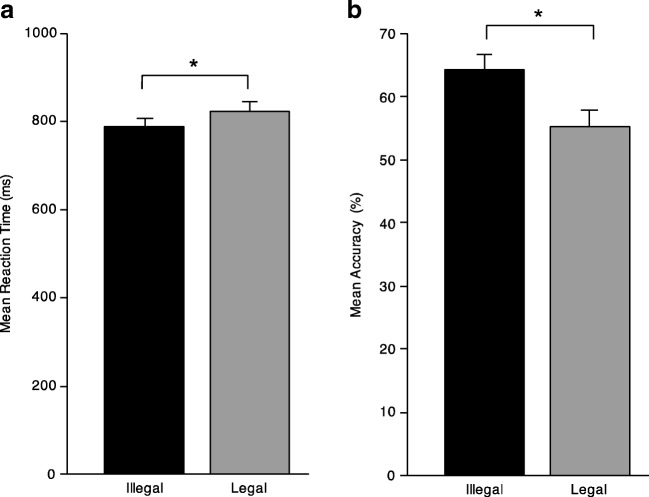
Behavioural results in the test phase: (**a**) reaction time and (**b**) accuracy, both collapsed across feedback condition. Error bars represent SEM*.* *p < .05

In terms of reaction times, there was a main effect of legality F(1,49) = 6.89, *p* = 0.012, ${\upeta}_{\mathrm{p}}^2$ = 0.123, with participants responding more quickly to illegal (*M*_Illegal_ = 786.92, *SD* = 136.23) than legal cards (*M*_legal_ = 822.10, *SD* = 150.60). However, there was not a main effect of feedback condition, nor an interaction (*p* ≥ 0.538). Similarly, with accuracy, we found a significant main effect of legality F(1,49) = 4.33, *p* = 0.043, ${\upeta}_{\mathrm{p}}^2$ = 0.081, but all other effects were nonsignificant (*p* ≥ 0.588), with participants responding more accurately to illegal (*M*_Illegal_ = 0.64, *SD* = 0.16) than legal cards (*M*_legal_ = 0.55, *SD* = 0.20), regardless of feedback condition (Fig. 5).

#### P3b

**Fig. 6:**
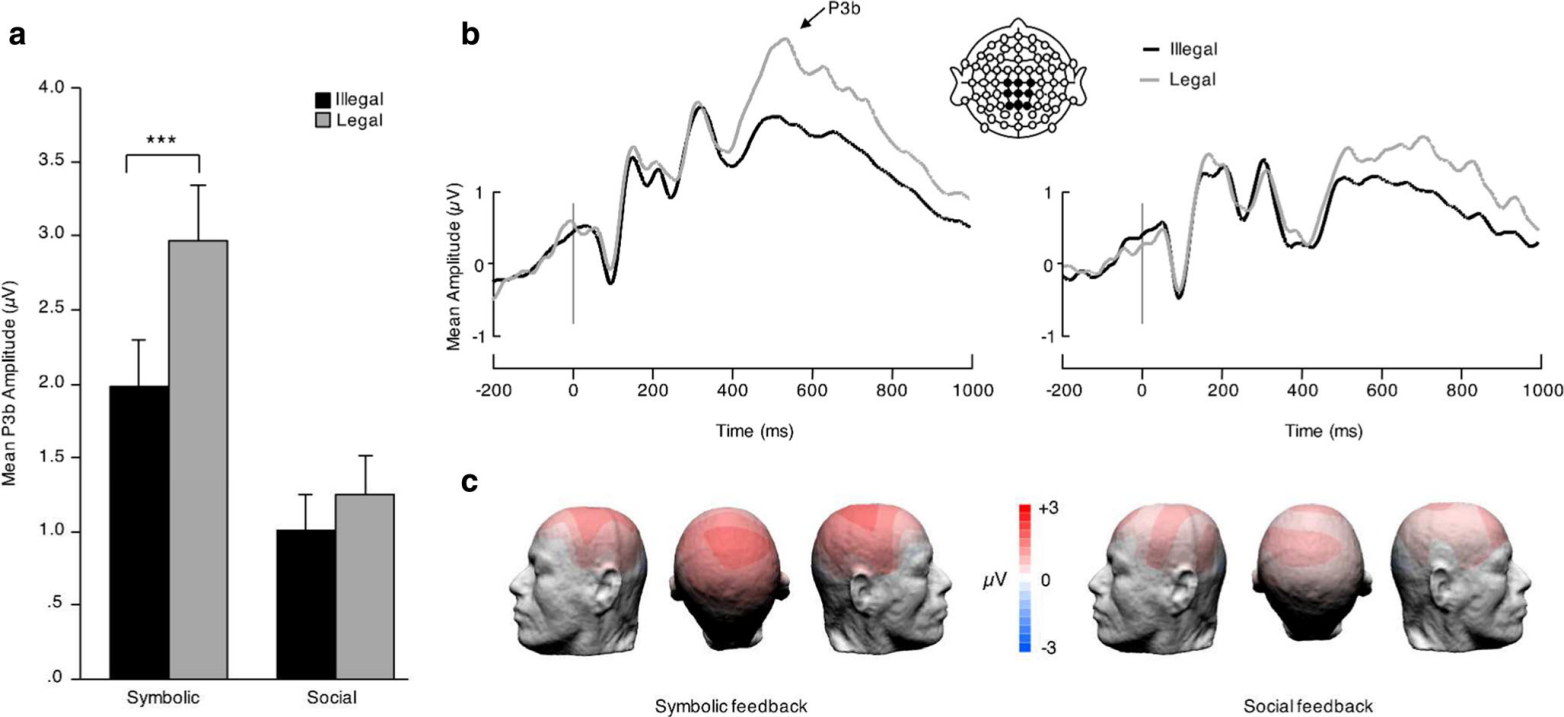
P3b Results. (**a**) Mean P3b amplitudes between 480-580 ms elicited over the centroparietal region (C1, Cz, C2, CP1, CPz, CP2, P1, Pz, P2) by legal (grey) and illegal (black) card triads in the symbolic (left) and social (right) feedback groups. Error bars indicate SEM. (**b**) P3b ERP waveforms elicited over the centroparietal region by legal (grey) and illegal (black) in the symbolic (left) and social (right) feedback groups. (**c**) P3b effect (legal minus illegal) topography by feedback group. ***p < 0.001

There were significant main effects of legality, F(1,45) = 30.29, *p* < 0.001, ${\upeta}_{\mathrm{p}}^2$ = 0.402, and feedback condition, F(1,45) = 10.49, *p* = 0.002, ${\upeta}_{\mathrm{p}}^2$ = 0.189, on P3b mean amplitudes. There also was a significant interaction between the two, F(1,45) = 11.04, *p* = 0.002, ${\upeta}_{\mathrm{p}}^2$ = 0.197. A simple effects analysis showed that the difference in amplitude between illegal (standard) and legal (deviant) trials was significant in the symbolic, F(1,45) = 39.80, *p* < 0.001,η^2^ = 0.457, but not the social feedback group, F(1,45) = 2.33, *p* = 0.134,η^2^ = 0.027 (Fig. 6).

### Across testing phases: P3b and fERN

**Fig. 7:**
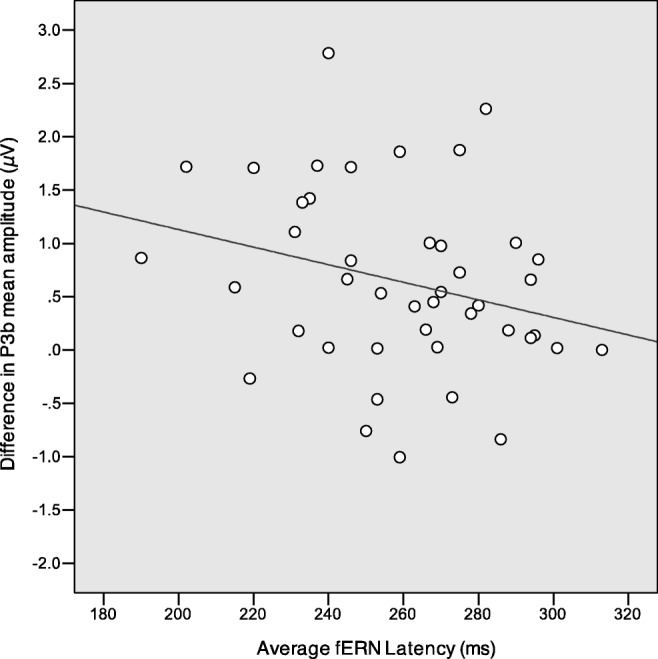
Relationship between fERN mean latency and P3b effect mean amplitude. Negative correlation between fERN mean latency and P3b effect (legal minus illegal) mean amplitude

Finally, in a subset of participants (N = 43) whose datasets were retained for both learning and test phases, we predicted that as the latency of the fERN increases, the difference between the oddball and standard stimuli (P3b effect) would decrease. We suggest that a delay in processing hindered feedback registration in the learning phase and lead to a weaker ERP response discriminating between illegal and legal stimuli at test. Analysis showed that there was indeed a significant negative correlation between these variables, *r*(41) = −0.27, *p* = 0.039, R^2^ = 0.07 (1-tailed), in the absence of an interaction with group, *p* > 0.1 (Fig. 7).

## Discussion

In the present study, we compared two groups of participants learning a new card game and receiving two different types of feedback: symbolic or social. We investigated the effect of feedback type on performance during learning and at test, using behavioural and ERP measures. In the learning phase, participants were more accurate overall for legal than illegal card triads, regardless of feedback type. Although we did not find any difference in mean ERP amplitude between groups, the fERN elicited by socially salient stimuli was significantly delayed (by ~20 ms) compared with that elicited by symbolic feedback, regardless of card triad legality. At test, participants showed greater accuracy for illegal than legal card combinations, suggesting that learning in the training phase had taken place. Furthermore, when collapsing across legality, participants in both groups displayed similar levels of accuracy, meaning that feedback type did not cause measurable differences in general performance. This being said, ERP differences between groups did manifest at test. Even though the paradigm we used was a non-traditional P3b design (i.e., participants were required to respond to all stimuli) a typical P3b effect was elicited in response to infrequent legal compared with frequent illegal stimuli in the symbolic group, while the social group failed to show a mean amplitude difference between conditions in the same time window. Finally, we found a correlation between fERN peak latency in the learning phase and P3b mean amplitude at test.

We expected socially salient feedback to enhance task performance (as suggested by results obtained by Hurlemann et al., 2010) but failed to find behavioural differences between participant groups. Nonetheless, brain responses differed between groups in both the learning and the testing phase, with the symbolic group displaying earlier fERN peaking time than the social group, as well as a significant P3b effect. Thus, whereas both groups learned the rule of the game to a similar extent, ERP measures indicate that the quality of learning differed depending on feedback type. This interpretation is consistent with the finding of a negative correlation between fERN latency and P3b amplitude: the greater the delay of negative feedback registration, the weaker the subsequent distinction between legal and illegal stimuli.

Thus, although feedback type does not differentially affect behavioural performance, socially relevant feedback appears to add cognitive noise affecting learning at the neurophysiological level. In that sense, the results echo those reported by Hu et al. (2015), who asked participants to sort 3-digit numbers according to arbitrary categories and gave feedback via either socially relevant (emotionally expressive faces) or symbolic (traffic light icons) stimuli. The authors found that social feedback impaired performance relative to symbolic feedback and that those learning via social feedback performed at comparable levels of accuracy as those learning based on symbolic feedback only after receiving a dose of intranasal oxytocin. The authors reasoned that the relative disadvantage afforded by emotional faces could be due to this stimulus type not being a customary form of feedback in Chinese culture. In other words, Chinese participants find emotive human faces disruptive during learning. Because we found a similar effect in our ERP data, the relative advantage of symbolic feedback may extend to western cultures. This effect may be explained by facial stimuli increasing cognitive load during difficult tasks (as in Doherty-Sneddon et al., 2002), which is consistent with other studies showing improvements in accuracy when participants avert the gaze from socially relevant stimuli during cognitive tasks (Glenberg et al., 1998; Phelps, Doherty-Sneddon, & Warnock, 2006). This result also concurs with the classic finding that the presence of others during cognitively demanding tasks can be detrimental to task performance (Bond & Titus, 1983). It is noteworthy that we used six different identities in the social feedback condition, thus incurring stimulus variability to a greater extent than that involved in the symbolic feedback group, given that symbolic feedback only varied in basic geometrical properties and lightness. The relatively greater diversity of stimuli in the social feedback version of the experiment therefore may have contributed to increasing the cognitive load in that condition and thus partly account for the pattern of difference found in the ERP data. Indeed, Hu et al. (2015) reported that using an emoticon instead of photographs of faces as feedback in the social feedback condition improves learning.

Note that the sharing of attributes between the shapes presented on cards and those used to provide feedback in the symbolic participant group could hardly account for this result. Symbolic feedback sometimes featured circles or squares, which could be green or red, attributes that could be represented in some cards. Whereas the semantic value of the symbolic shapes was entirely based on colour, unambiguous (i.e., green = correct, red = incorrect), and binary in nature, the shape and colour attributes of shapes on cards were entirely arbitrary and only had value when considered across cards. Indeed, there was no detrimental effect of the overlap in attributes between card shapes and symbolic feedback shapes, thus not causing any measurable consequences in this study.

Our task deliberately engaged spatial and abstract-reasoning capabilities. Thus, it was cognitively demanding, which may explain why we did not find social feedback to have a facilitatory effect on task performance but rather tend to cause shallower learning, indexed by P3b amplitudes. We provided electrophysiological evidence that socially salient feedback—when the task at hand is abstract and relatively complex—is less conducive to facilitating implicit learning, as evidenced by a delayed fERN. Participants in this feedback group also showed a reduced P3b effect, reflecting a decreased ability to distinguish between rare legal stimuli and more frequent illegal stimuli. Our results thus support recommendations that during difficult cognitive tasks, people should avoid looking at other individuals to increase task accuracy (Phelps et al., 2006). However, future research is needed to generalise this finding to other learning contexts (e.g., socially relevant tasks) and investigate whether it is the social quality of the stimuli or its informativeness in the task context that affects learning quality on the neurophysiological level.

We employed a type of negative social feedback different from that used in previous studies (Hu et al., 2015; Hurlemann et al., 2010; Mihov et al., 2010). The latter experiments used “angry” faces as negative feedback in social conditions, whereas we elected to use “sad” faces. One could argue that sadness does not convey negative feedback in response to an error in learning contexts as efficiently as frowning or anger. This may have resulted in a slightly different emotional context, thus limiting the validity of direct comparisons between studies.

Another point to note was the use of unequal number of possible combinations of the four shape dimensions per level of difficulty at test (see Stimuli section). In the learning phase, we ensured that the different combinations had equal probability of presentation to offer participants a chance to learn all possible combination types. However, during the test phase, we allowed the combinations to occur at their natural frequency. For example, there are many possible combinations of illegal trials in which the rule is violated for one stimulus dimension only compared with the case of violations affecting all dimensions simultaneously. Future studies will determine whether the local frequency of each difficulty level has a measurable impact on detecting legal combinations amongst illegal ones. Indeed, once a rule is learned, it is unclear how the diversity of test stimuli affects performance, because the criterion acquired during the learning phase is binary in nature.

## Conclusions

This study investigated the role of feedback during an implicit-intentional learning task. Contrary to our hypotheses drawn from previous behavioural studies, we found that symbolic feedback was more effective than social feedback, as demonstrated by a delayed fERN in the social group during learning, and lower ability to distinguish between card combinations that complied to an implicitly learnt rule and those that did not at test, as indexed by a neurophysiological index of target detection (P3b). We suggest that the social salience of feedback may interfere with the learning process, at least when the rule to be learnt is abstract and relatively complex. Such effects need to be further investigated in experiments directly manipulating task complexity in social versus nonsocial feedback context and characterise the importance of the type of social feedback received.
